# Comprehensive analysis of the cuproptosis-related model to predict prognosis and indicate tumor immune infiltration in lung adenocarcinoma

**DOI:** 10.3389/fonc.2022.935672

**Published:** 2022-10-20

**Authors:** Minle Wu, Jie Bao, Youfeng Lei, Shuai Tao, Qiurong Lin, Liang Chen, Yinpeng Jin, Xiaohong Ding, Yufeng Yan, Ping Han

**Affiliations:** ^1^ Department of Laboratory Medicine, Shanghai Public Health Clinical Center, Fudan University, Shanghai, China; ^2^ Department of Pharmacy, Anhui Provincial Corps Hospital of Chinese People’s Armed Police Forces, Hefei, China; ^3^ Department of Pharmacy, Shanghai Public Health Clinical Center, Fudan University, Shanghai, China; ^4^ Department of Research Unit, Shanghai Public Health Clinical Center, Fudan University, Shanghai, China; ^5^ Department of Ophthalmology, Shanghai Eye Diseases Prevention and Treatment Center, Shanghai Eye Hospital, Shanghai, China; ^6^ Department of Liver Disease Center, Shanghai Public Health Clinical Center, Fudan University, Shanghai, China

**Keywords:** lung adenocarcinoma, cuproptosis, tumor microenvironment, immunotherapy, immune infiltrates

## Abstract

**Background:**

Cuproptosis is a novel form of programmed cell death termed as Cu-dependent cytotoxicity. However, the roles of cuproptosis-associated genes (CAGs) in lung adenocarcinoma (LUAD) have not been explored comprehensively.

**Methods:**

We obtained CAGs and utilized consensus molecular clustering by “non-negative matrix factorization (NMF)” to stratify LUAD patients in TCGA (N = 511), GSE13213 (N = 117), and GSE31210 (N = 226) cohorts. The ssGSEA and CIBERSORT algorithms were used to evaluate the relative infiltration levels of immune cell types in tumor microenvironment (TME). The risk score based on CAGs was calculated to predict patients’ survival outcomes.

**Results:**

We identified three cuproptosis-associated clusters with different clinicopathological characteristics. We found that the cuproptosis-associated cluster with the worst survival rates exhibited a high enrichment of activated CD4/8^+^ T cells. In addition, we found that the cuproptosis-associated risk score could be used for patients’ prognosis prediction and provide new insights in immunotherapy of LUAD patients. Eventually, we constructed a nomogram-integrated cuproptosis-associated risk score with clinicopathological factors to predict overall survival in LUAD patients, with 1-, 3-, and 5-year area under curves (AUCs) being 0.771, 0.754, and 0.722, respectively, all of which were higher than those of the TNM stage.

**Conclusions:**

In this study, we uncovered the biological function of CAGs in the TME and its correlations with clinicopathological parameters and patients’ prognosis in LUAD. These findings could provide new angles for immunotherapy of LUAD patients.

## Introduction

Lung cancer is still one of the most common malignant tumors and the main cause of cancer-related deaths worldwide (1, 2). Non-small cell lung cancer (NSCLC) accounts for 85% of lung cancer cases, which can be further divided into three subtypes: lung adenocarcinoma (LUAD), squamous cell carcinoma, and large cell carcinoma (3, 4). LUAD is presently the most common histological type of NSCLC (5), which is related to factors such as smoking, drinking, and metabolic disorder (3, 6, 7). Although considerable progress has been made in the comprehensive treatment of LUAD (surgery, chemotherapy, radiotherapy, targeted therapy, and immunotherapy), the survival rate of LUAD patients is still relatively low (8, 9). Accumulating studies have shown that the traditional histological classification of LUAD has limitations for treatment due to its high heterogeneity and tumor complexity (10–12). Therefore, increasing molecular subtypes are being studied to guide treatment (13–15). A better understanding of the relationship between tumor microenvironment disturbance caused by gene changes and the prognosis of LUAD patients is very important for the development of new therapeutic targets.

There are many predetermined and accurately controlled programmed cell deaths during the development of multicellular organisms, such as apoptosis (16), necroptosis (17), pyroptosis (18), and ferroptosis (19). Copper is a trace element in the human body (20). The concentration of copper ion in cells is maintained at a very low level through active homeostasis mechanisms (21). Once it exceeds the threshold, copper will become toxic and lead to cell death (22). However, the mechanism of copper-induced cytotoxicity is still unclear. Recently, researchers have confirmed that the copper-dependent controlled cell death mode is a new cell death mode different from the known cell death mechanism, which is named cuproptosis (23, 24). More importantly, the study further identified which cells are more vulnerable to cuproptosis, which developed new potential treatments for cancer.

The homeostasis and evolution of the tumor microenvironment (TME) are controlled by cross talk within and between all cell compartments, including malignant cells, endothelial cells, stromal cells, and immune cells (25). This complex interaction usually involves the regulation of programmed cell death mode and extracellular metabolites. Research evidence supports that the TME plays a key role in treatment response and patient outcome, which reflects the tumor immune response and predicts the therapeutic benefit (26). In addition to the impact on immunotherapy, the TME also affects the efficiency of chemotherapy and radiotherapy through the original characteristics of the TME and the treatment-induced response in the TME (27). The number of infiltrated T cells, macrophages, and cancer-related fibroblasts in the TME is related to the prognosis of patients with various cancers, including lung cancer, urothelial cancer, and esophageal cancer. Therefore, the characterization of the TME is helpful to develop prognostic and predictive biomarkers and identify new therapeutic targets.

In this study, we will comprehensively explore the important role of cuproptosis in LUAD, so as to clarify the significance of cuproptosis as an important biomarker for the prognosis, molecular subtypes, and infiltration cell characteristics of the TME in LUAD patients.

## Methods

### RNA expression datasets

TCGA-LUAD datasets were curated from UCSC Xena (https://xenabrowser.net/datapages/GDC TCGA Lung Adenocarcinoma (LUAD)) (N = 511). Somatic mutation data were downloaded from https://portal.gdc.cancer.gov/repository. The gene expression profiles of the GSE13213 (N = 117) (28) and GSE31210 (N = 226) (29) datasets were downloaded from the GEO database (http://www.ncbi.nlm.nih.gov/geo/).

### Cuproptosis-related genes included for analysis

Ten cuproptosis-related genes were retrieved from a previous study. The description of cuproptosis-related genes is shown in **Table S1**.

### Principal component analysis and consensus molecular clustering by “non-negative matrix factorization”

Principal component analysis (PCA) was performed using highly variable genes identified by the SEURAT function “FindVariableGenes()”. NMF is widely used for clustering high-dimensional data sets in computational biology (30). Cuproptosis-related molecular clusters were identified by consensus clustering the “NMF” function in TCGA-LUAD cohort. The description of clusters of LUAD patients is shown in **Table S2**.

### Immune analysis

We performed the CIBERSORT deconvolution approach to evaluate the relative abundance of 22 tumor-infiltrating immune cells (TIICs). Next, gene signatures of immune cells from the research of Charoentong were used for calculating the immune infiltration-related score by single-sample gene set enrichment analysis (ssGSEA).

### Somatic mutation analyses

Somatic mutations presented in VarScan file format were downloaded from https://portal.gdc.cancer.gov/repository. Copy number variation files were curated from UCSC Xena online.

### DLD gene expression level by RT-PCR and immunohistochemistry

We obtained normal and cancer frozen tissues and formalin-fixed paraffin-embedding (FFPE) samples from 20 LUAD patients in our center and detected the DLD gene transcriptome expression level by RT-PCR and the DLD protein level by immunohistochemistry (IHC) [anti-DLD antibody (A13296, ABclonal)].

DLD sense 5′-CTCATGGCCTACAGGGACTTT-3′;

anti-sense 5′-GCATGTTCCACCAAGTGTTTCAT-3′;

β-actin sense 5′-CGCGAGAAGATGACCCAGAT-3′;

anti-sense 5′-GGGCATACCCCTCGTAGATG-3′

### Construction of the cuproptosis-related prognostic risk score

We first performed differentially expressed gene (DEG) analyses in each NMF cluster and obtained 499 genes referred to as cuproptosis phenotype-related genes. After combination of the 10 cuproptosis-related genes, univariate Cox regression analysis was performed to identify those linked to overall survival. Then, we totally performed 1,000 iterations and included five gene groups for further screening, as previously described. A gene model with 16 genes showed the highest frequencies of 459 compared to other four-gene models. Finally, 16 genes were used to generate the gene signature for calculating risk score, which was calculated as follows:


Risk score = Σ(Expi * coefi)

By setting the median value of risk_score as the threshold, patients in different cohorts could be divided into high- and low-risk groups. The subsequent receiver operating characteristic (ROC) and Kaplan–Meier (K–M) survival curves were plotted according to high- and low-risk groups. The values of coefficient of 16 genes are shown in **Table S3**.

### Prognostic nomogram for LUAD and area under the curve

The influence of variables on overall survival (OS) of LUAD patients was determined by univariate and multivariate Cox regression analyses. Based on multivariate Cox regression analysis, a nomogram was developed to integrate tumor stage and risk score to predict the prognosis of LUAD patients. The prediction ability of the nomogram is evaluated by the AUC.

### Statistical analyses

Statistical analysis was performed using R (version 4.0.0) and GraphPad Prism (version 7.04). The Wilcox test, log-rank test, and Kruskal–Wallis H test were performed in this study. Detailed descriptions of statistical tests are specified in the figure legends.

## Results

### Genetic variation of cuproptosis-associated genes in LUAD

**Figure 1:**
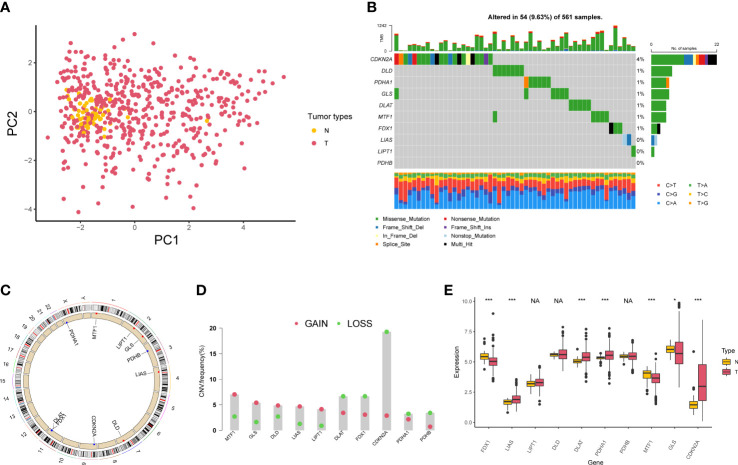
Genetic variation of cuproptosis-associated genes in lung adenocarcinoma. **(A)** Principal component analysis of normal and tumor samples. **(B)** Mutation of 10 cuproptosis-associated genes in lung adenocarcinoma. **(C)** Locations of copy number variation alterations in 10 cuproptosis-associated genes on 23 chromosomes. **(D)** Copy number variation frequency of 10 cuproptosis-associated genes in TCGA-LUAD samples. **(E)** The boxplot shows the expression of 10 cuproptosis-associated genes between normal and tumor tissues. *P < 0.05; ***P < 0.001; NA, no difference in statistics.

A total of 10 genes associated with cuproptosis were obtained according to previous research (23), which were named as cuproptosis-associated genes (CAGs) in this study. PCA indicated that CAGs could discriminate tumor tissues from normal samples in TCGA-LUAD cohort (**Figure 1A**). Somatic mutations of CAGs were observed in 54 of 561 samples (**Figure 1B**). Copy number variation (CNV) analyses are shown in **Figures 1C, D**. According to the expression level of CAGs in LUAD samples, we found that LIAS was highly expressed in the tumor, consistent with its CNV amplification. FDX1 was downregulated in the tumor, in line with its CNV depletion (**Figure 1E**). In order to analyze the translational levels of CAGs, the Human Protein Atlas (HPA) database was used (**Figure S1**). We found that the expression intensity and quantity of DLD, DLAT, PDHB, MTF1, and CDKN2A in LUAD tissue were higher than that in normal lung tissue.

### Cuproptosis-associated classifications in LUAD

**Figure 2:**
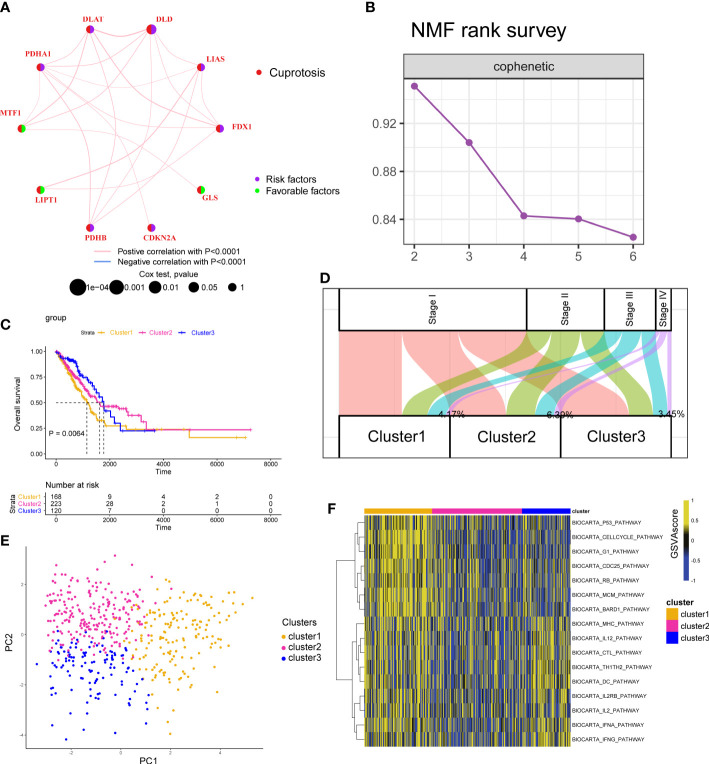
Cuproptosis-associated classifications in lung adenocarcinoma. **(A)** A network describes the connection and prognostic values of 10 cuproptosis-associated genes. **(B)** The non-negative matrix factorization (NMF) rank survey was shown. The optimal number of clusters: rank = 3. **(C)** K–M survival plots according to NMF clusters. The P value was determined by the log-rank test. **(D)** The distribution plot shows the composition of clinicopathological features of three NMF clusters. **(E)** Principal component analysis shows the distribution of three NMF clusters. **(F)** Corresponding pathway activities of three NMF clusters.

We first displayed the connections and prognostic values of 10 CAGs in **Figure 2A**. To clearly understand the correlation between CAGs and clinical prognosis, we utilized the COX and Kaplan–Meier (K–M) survival analyses. The results indicated that only DLD had significant prognostic values in both of the two analyses (**Figure S2**). RT-PCR and IHC results showed that the expression level of the DLD gene in LUAD patients was higher than that in normal tissues (**Figure S3**). Then, based on the expression of CAGs, tumor samples in TCGA-LUAD and GSE13213 cohorts were classified into three clusters (**Figures 2B**; **S4A**, **S5A**) with different survival rates (**Figures 2C**; **S5B**). We observed that cluster 1 exhibited the worst survival rates, of which 4.17% samples were clustered into stage IV diseases (**Figures 2D**; **S5C**). PCA showed that CAGs could divide tumor samples into these three clusters (**Figure 2E**). To delineate the biological function between different clusters, we performed pathway analysis and found that cancer-related pathways such as cell cycle and immune-related pathways such as BIOCARTA_IFNG_PATHWAY and BIOCARTA_TH1TH2_PATHWAY were mainly enriched in cluster 1 (**Figures 2F**; **S4B**, **S5D**).

### Analyses of tumor microenvironment of cuproptosis-associated clusters

**Figure 3:**
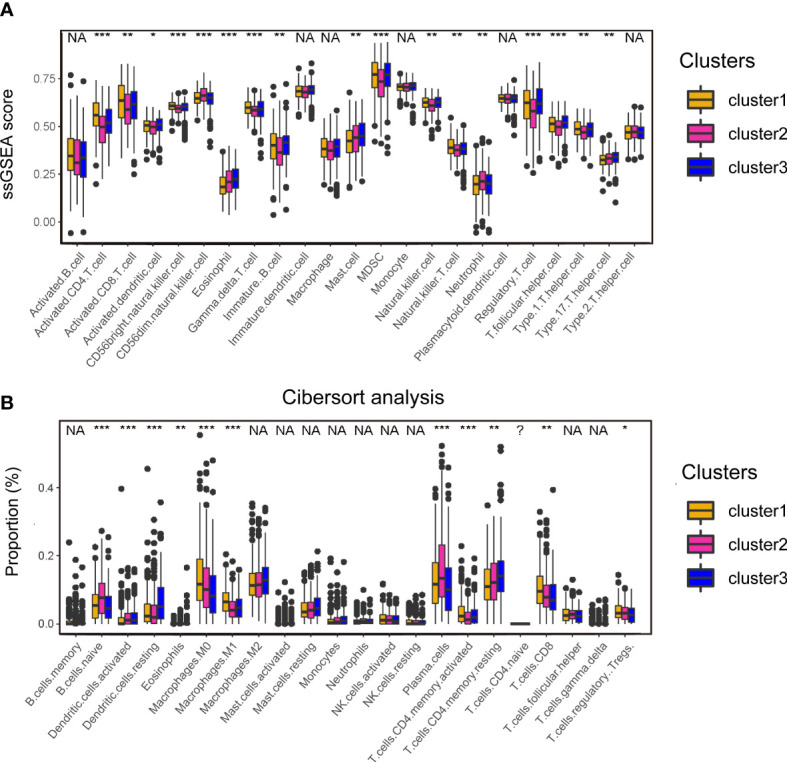
Analyses of the tumor microenvironment of cuproptosis-associated clusters. **(A)** ssGSEA of immune cells in three clusters. The statistical difference of three clusters was compared by the Kruskal–Wallis H test. *P < 0.05; **P < 0.01; ***P < 0.001; NA, no difference in statistics. **(B)** Boxplot shows the proportion of immune cells calculated by CIBERSORT analysis. The statistical difference of three clusters was compared by the Kruskal–Wallis H test. *P < 0.05; **P < 0.01; ***P < 0.001; NA, no difference in statistics.

To understand the heterogenicity of the tumor microenvironment of three cuproptosis-associated clusters, we performed immune-related analyses including ssGSEA and CIBERSORT. Here, we demonstrated that MDSCs and regulatory T cells (Tregs), both of which were correlated with the suppressive microenvironment, were mainly enriched in cluster 1 (**Figures 3A**; **S6B**). Interestingly, we observed that antitumor-related immune cells such as activated CD4/8^+^ T cells (**Figures 3A**; **S6A**) and CD8^+^ T cells (**Figures 3B**; **S6A**) were predominantly enriched in cluster 1.

### Development of cuproptosis-associated risk score

**Figure 4:**
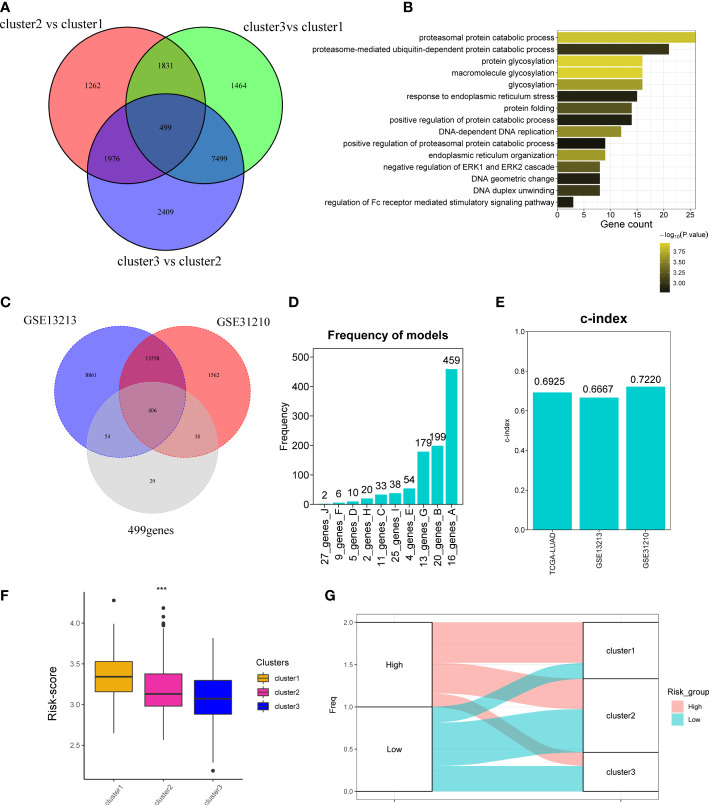
Development of cuproptosis-associated risk score. **(A)** Venn plot shows the differentially expressed genes called as cuproptosis phenotype-related genes. **(B)** GO function of differentially expressed genes. **(C)** Venn plot shows the overlapped genes of GSE13213, GSE31210, and 499 differentially expressed genes detected in TCGA-LUAD cohort. **(D)** Frequency models calculating the gene models. **(E)** Bar plot shows the c-index of two datasets. **(F)** Boxplot shows the risk_score of three clusters. **(G)** The distribution of three clusters in the high- and low-risk groups. *P < 0.05; **P < 0.01; ***P < 0.001.

The above analyses were mainly based on 10 CAGs; it seems to be few genes about cuproptosis. To further explore whether there are more genes related to cuproptosis, we performed DEG analysis as previously reported and obtained 499 genes (**Figure 4A**). Analyses of biological function showed that 499 genes were related to protein catabolic process and glycosylation (**Figure 4B**). We finally recognized 499 genes as cuproptosis phenotype-related genes. Then, we would like to investigate the correlation between cuproptosis phenotype-related genes and clinical prognosis. Three datasets were curated for further analyses, namely, TCGA-LUAD, GSE13213, and GSE31210 cohorts. We found that 406 of 499 genes could be detected in three datasets (**Figure 4C**) and combined them with 10 CAGs for construction of the risk score model. By 1,000 iterations, we observed that a cluster of 16 genes presented the highest frequency (459 times; **Figure 4D**), and they were chosen for calculating the risk score. The c-indexes of risk score in each dataset were 0.6925, 0.6667, and 0.7220 (**Figure 4E**). Afterward, we found that high risk score was positively correlated with cluster 1, in line with the fact that cluster 1 had the worst survival rates (**Figures 4F, G**).

### Predictive ability of cuproptosis-associated risk score for prognosis

**Figure 5:**
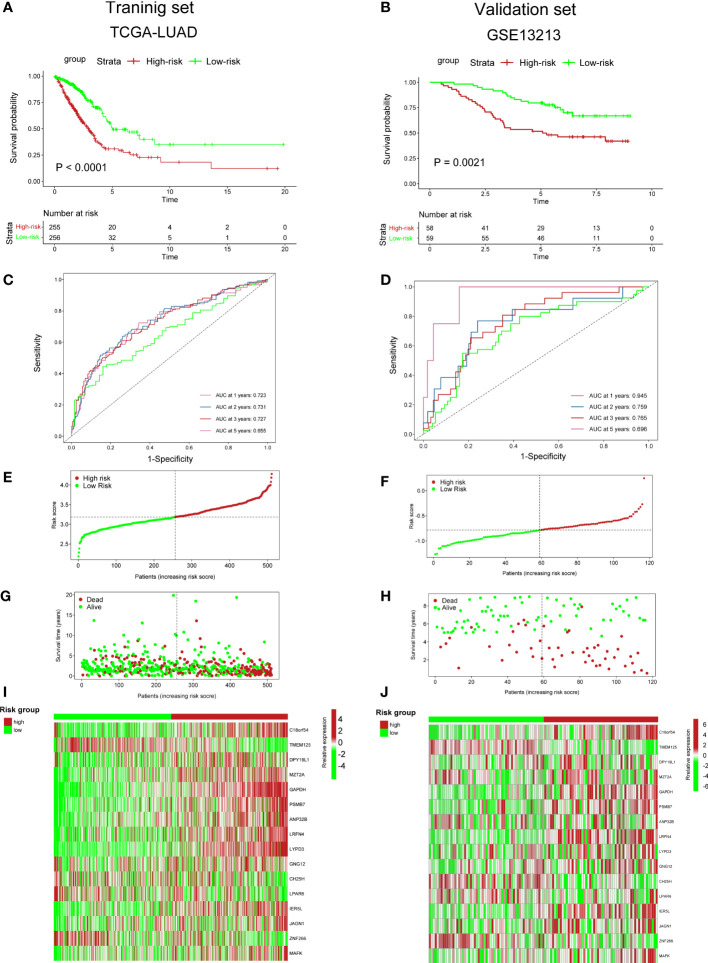
Predictive ability of cuproptosis-associated risk score for prognosis. **(A, B)** K–M survival curves. P value was determined by the log-rank test. **(C, D)** ROC curves show AUC values of risk_score. **(E, F)** Distribution plot shows risk_score. **(G, H)** Distribution plot shows the correlation between risk_score and patients’ survival status. **(I, J)** The heatmap plot shows the expression of 36 genes in high- and low-risk groups.

To validate the accuracy of our cuproptosis-associated risk score for predicting patients’ prognosis, we performed K–M survival analysis and found that in the training (TCGA-LUAD), validation (GSE13213), and external datasets (GSE31210), the high-risk group exhibited a worse survival rate (**Figures 5A, B**; **S7A**). The AUC values of survival rates at 1, 2, 3, and 5 years in three cohorts were over 0.6 (**Figures 5C, D**; **S7B**), suggesting that our cuproptosis-associated risk score could predict prognosis precisely. The distribution plot also indicated that the cuproptosis-associated risk score was increasing with the rate of death (**Figures 5E–H**; **S7C** **, D**). Finally, the expression of 16 genes used for the cuproptosis-associated risk score model in high- and low-risk groups is shown in **Figures 5I, J**; **S7E**.

### Analyses of TME in high- and low-risk groups

**Figure 6:**
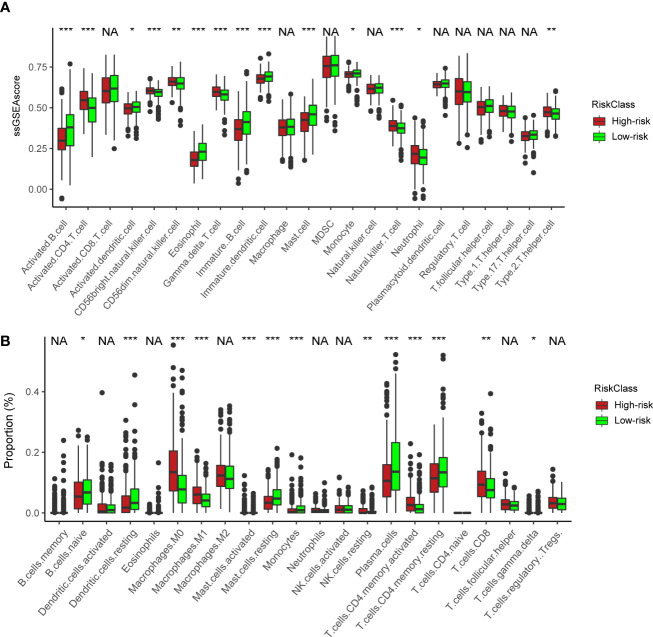
Immune characteristics between the high- and low-risk groups. **(A)** ssGSEA of immune cells in high- and low-risk groups. The statistical difference of three clusters was compared by the Kruskal–Wallis H test. *P < 0.05; **P < 0.01; ***P < 0.001. **(B)** Boxplot shows the proportion of immune cells calculated by CIBERSORT analysis. The statistical difference of three clusters was compared by the Kruskal–Wallis H test. *P < 0.05; **P < 0.01; ***P < 0.001; NA, no difference in statistics.

We next utilized ssGSEA and CIBERSORT to understand the TME infiltration in high- and low-risk groups. The results showed that activated CD4^+^ T cells (**Figure 6A**) and CD8^+^ T cells (**Figure 6B**) were mainly enriched in the high-risk group. In previous analyses, the high-risk group was predominantly clustered into NMF cluster 1 (**Figure 4G**). Therefore, combined with the similar immune infiltration (CD4/8^+^ T cells) of cluster 1, we postulated that activated CD4/8^+^ T cells could be targeted immune cells to improve anticancer immunotherapy.

### A nomogram constructed for predicting patients’ prognosis

**Figure 7:**
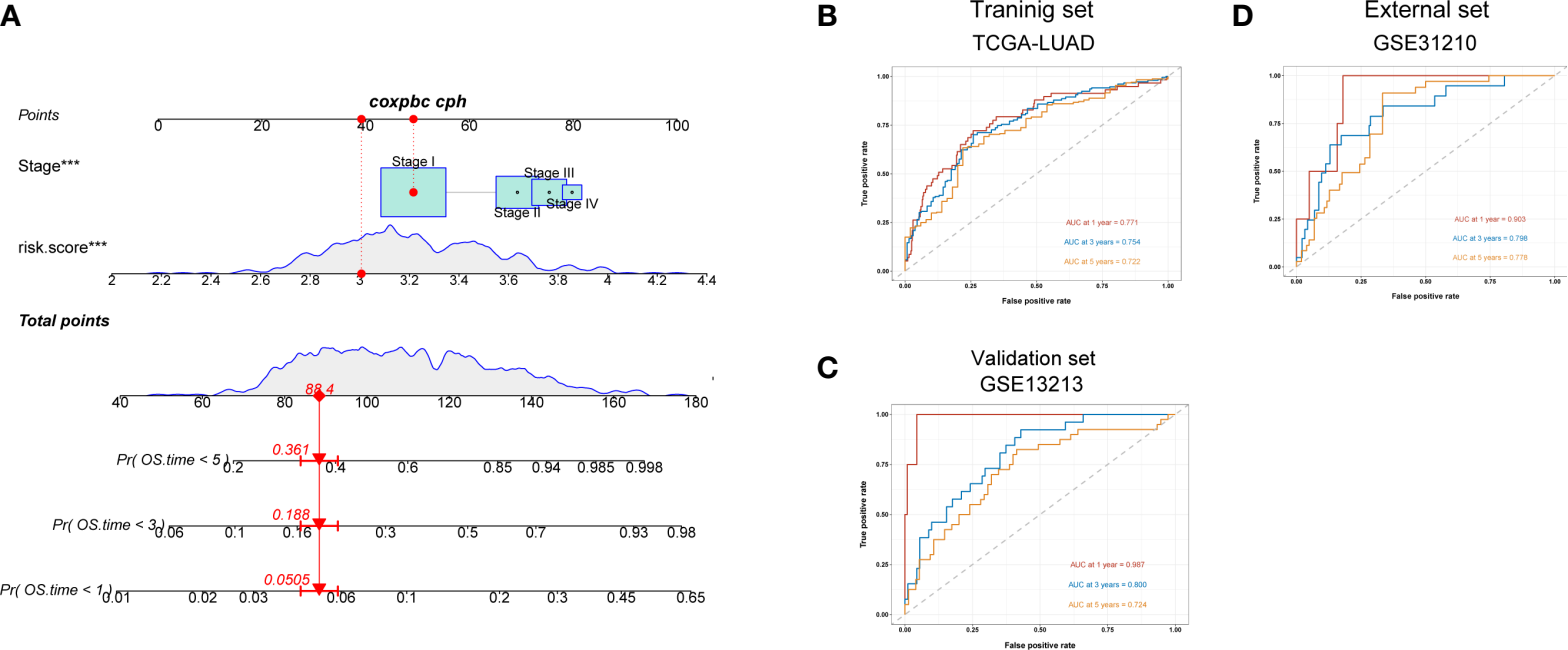
A nomogram constructed for predicting patients’ prognosis. **(A)** Nomogram for predicting the 1-, 3-, and 5-year OS of lung adenocarcinoma patients in the training set. **(B–D)** ROC curves for predicting the 1-, 3-, and 5-year ROC curves in the training (TCGA), validation (GSE13213) cohort, and external cohort (GSE31210).

We next constructed a nomogram to predict overall survival in LUAD patients (**Figure 7A**). Here, we observed that AUC values of nomogram at 1, 3, and 5 years of the training set were 0.771, 0.754, and 0.722, respectively (**Figure 7B**), all of which were higher than the AUC values of the TNM stage at 1, 3, and 5 years (**Figure S8D**). Similarly, the AUC values of nomogram in validation (**Figure 7C**) and external sets (**Figure 7D**) were also higher than those of the disease stage (**Figures S8E, F**) at 1, 3, and 5 years. The calibration plots of the nomogram are displayed in **Figures S8A–C**. All these findings indicated that our nomogram showed an advantage in predicting LUAD patients’ prognosis.

## Discussion

Due to the tumor heterogeneity of LUAD, the overall survival rate of LUAD patients is limited (1). In recent years, significant progress has been made in the treatment of LUAD, but owing to the limitations of traditional histological classification in guiding tumor treatment, accurately identifying LUAD molecular subtypes is increasingly important. Previous studies have been carried out in this field, such as immunophenotyping (31) and metabolic phenotype (32), but there are still considerable limitations in each study. Therefore, a more in-depth exploration of LUAD is urgently needed to improve the survival rate of patients.

In our study, we found the genetic variation of cuproptosis-associated genes in LUAD. The 10 CAGs can divide LUAD patients into three different molecular subtypes, which are significantly related to the patient prognosis. Further pathway analysis showed that different cuproptosis-associated clusters were closely related to different tumor-related pathways and immune pathways. Therefore, we developed a cuproptosis-associated risk score and validated it in different data sets to evaluate its prognostic ability. Meanwhile, by analyzing the TME in high- and low-risk groups, we found that activated CD4^+^ T cells and CD8^+^ T cells were mainly enriched in high-risk groups. Therefore, we hypothesized that activated CD4^+^/8^+^ T cells could be used as targeted immune cells to improve anticancer immunotherapy. Finally, we combined the cuproptosis-associated risk score with the clinicopathological factors to construct a nomogram to predict the overall survival rate of LUAD patients. This nomogram showed unique advantages in predicting the prognosis of LUAD patients. Admittedly, the nomogram needs to be validated in large sample size prospective clinical trials before routinely clinical application.

Cuproptosis is a hot topic in modern medicine (33). Cuproptosis is different from other programmed cell deaths. It does not depend on the apoptosis pathway. The accumulation of copper destroys mitochondrial function, and the destruction of lipoacylase inhibits copper toxicity. The role of cuproptosis-related genes in LUAD has not been studied. In our study, we constructed a gene prognostic model related to cuproptosis in LUAD. This is the first prognostic nomogram related to cuproptosis to evaluate the prognosis of LUAD patients, which has objective clinical value. Meanwhile, we found the characteristics of the TME between high- and low-risk LUAD patients according to cuproptosis-associated risk scores, especially the infiltration of immune cells, which provides potential value for immunotherapy of high-risk patients and also confirms the important role of cuproptosis in the immune microenvironment. Further studies on relevant molecular mechanisms between cuproptosis and the TME are urgently needed.

Most of the 10 genes associated with cuproptosis are closely related to cancers. DLAT is one of three mitochondrial proteins found to be upregulated in eight of 11 gastric cancer cell lines (34). It exists in the inner membrane of the mitochondria and plays a role in the decomposition of pyruvate into acetyl CoA. It is found that DLAT protein may be one of the potential drug targets in mitochondria, which provides a theoretical basis for drug therapy designed for mitochondria (35). CDKN2A has been proved to play an important function in various cancers. A meta-analysis suggests that CDKN2A hypermethylation may be a predictor of poor prognosis in patients with colorectal cancer (36). It was found that the frequent deletion of CDKN2A was related to the downregulation of CDKN2A in lung cancer. Knockout of CDKN2A significantly stimulated cell proliferation, invasion, and migration (37). Zhang et al. (38) found that FDX1 can affect the prognosis of LUAD patients. Further studies found that knockout of FDX1 neither inhibited the growth of tumor cells nor induced apoptosis or abnormal cell cycle distribution. However, FDX1 can promote the production of ATP. In addition, FDX1 is closely related to glucose metabolism, fatty acid oxidation, and amino acid metabolism (38). Metal regulatory transcription factor 1 (MTF1) is a conserved metal binding transcription factor that binds to conserved DNA sequence motifs in eukaryotes, which is called metal response element (39). MTF1 binds to chromatin in the promoter region of the myoblast gene, which is stimulated by the addition of copper. These findings revealed an unexpected mechanism by which copper and MTF1 regulate gene expression during myoblast differentiation (39). GLS also plays an important role in various tumors. Tong et al. (40) demonstrated that GLS was highly expressed in human pancreatic ductal adenocarcinoma (PDAC) specimens and correspondingly upregulated the glutamine dependence on PDAC cell proliferation. The results of Mukha et al. (41) showed that GLS-driven glutamine lysis is a prognostic biomarker and therapeutic target of radiation sensitization of prostate cancer.

There are some limitations. First, a large number of LUAD samples are needed to verify the stability of this new molecular typing and nomogram. Second, the molecular function of cuproptosis-related genes needs further basic experiments. The relationship between cuproptosis and immunity needs further experimental verification.

## Conclusion

In conclusion, we comprehensively explored the cuproptosis-associated molecular subtypes and identified their correlations with TME cell-infiltrating characteristics. These integrated analyses will contribute to understanding the TME infiltration based on cuproptosis and provide an interesting insight into immunotherapeutic efficacy.

## Data availability statement

The original contributions presented in the study are included in the article/**Supplementary Material**. Further inquiries can be directed to the corresponding authors.

## Ethics statement

The Ethical Committee and Institutional Review Board of Shanghai Public Health Clinical Center reviewed and approved this study protocol.

## Author contributions

PH and MW had the idea for this study. MW, JB, YL, QL, ST, LC, YJ, XD, YY, and PH supervised the acquisition of the data. MW, JB, YL, YY, and PH undertook the statistical analysis. JB, YL, and YY provided statistical advice. All authors contributed to interpretation of the results. MW and PH wrote the article. MW, YY, and PH revised the article, and other authors contributed to the content. All authors approved the final version of the manuscript, including the authorship list.

## Funding

This study was funded by the National Natural Science Foundation of China (Grant No. 82000594).

## Acknowledgments

The authors would like to thank the GEO and TCGA databases for the support.

## Conflict of interest

The authors declare that the research was conducted in the absence of any commercial or financial relationships that could be construed as a potential conflict of interest.

## Publisher’s note

All claims expressed in this article are solely those of the authors and do not necessarily represent those of their affiliated organizations, or those of the publisher, the editors and the reviewers. Any product that may be evaluated in this article, or claim that may be made by its manufacturer, is not guaranteed or endorsed by the publisher.

## Abbreviations

CAG, cuproptosis-associated gene; LUAD, lung adenocarcinoma; TME, tumor microenvironment; NSCLC, non-small cell lung cancer; TIIC, tumor-infiltrating immune cell; ssGSEA, single-sample gene set enrichment; DEG, differentially expressed gene; K–M, Kaplan–Meier; PCA, principal component analysis; CNV, copy number variation; Tregs, regulatory T cells; PDAC, pancreatic ductal adenocarcinoma.
