# Supplementary material for: Comprehensive analysis of the cuproptosis-related model to predict prognosis and indicate tumor immune infiltration in lung adenocarcinoma
Source: Front Oncol. 2022 Oct 20;12:935672. doi: 10.3389/fonc.2022.935672 (PMC9631493; doi:10.3389/fonc.2022.935672)
Supplement: Supplementary file 4 [file Table_4.docx]

Raw data for “Comprehensive Analysis of Cuproptosis-Related Model to Predict Prognosis and Indicate Tumor Immune Infiltration in Lung Adenocarcinoma” can be download from the following website: https://www.jianguoyun.com/p/DZDFpb4QrvbFChj6vr0EIAA
