# Supplementary material for: Comprehensive analysis of the cuproptosis-related model to predict prognosis and indicate tumor immune infiltration in lung adenocarcinoma
Source: Front Oncol. 2022 Oct 20;12:935672. doi: 10.3389/fonc.2022.935672 (PMC9631493; doi:10.3389/fonc.2022.935672)
Supplement: Supplementary file 5 [file DataSheet_1.doc]

**Supplemental file**

**Comprehensive Analysis of Cuproptosis-Related Model to Predict Prognosis and Indicate Tumor Immune Infiltration in Lung Adenocarcinoma**

Minle Wu^1*^, Jie Bao^2*^, Youfeng Lei^3*^, Qiurong Lin^4^, Shuai Tao^5^, Yinpeng Jin^6^, Xiaohong Ding^3^, Yufeng Yan^3,#^, Ping Han^3,#^

^1^Department of Laboratory Medicine, Shanghai Public Health Clinical Center, Fudan University, Shanghai, China;

^2^Department of Pharmacy, Anhui Provincial Corps Hospital of Chinese People’s Armed Police Forces, Hefei, China;

^3^Department of Pharmacy, Shanghai Public Health Clinical Center, Fudan University, Shanghai, China;

^4^Department of Ophthalmology, Shanghai Eye Diseases Prevention & Treatment Center, Shanghai Eye Hospital, Shanghai, China;

^5^Department of Research Unit, Shanghai Public Health Clinical Center, Fudan University, Shanghai, China;

^6^Department of Liver Disease Center, Shanghai Public Health Clinical Center, Fudan University, Shanghai, China.

^#^ Corresponding authors: Dr. Ping Han, E-mail address: [hanp_elf@163.com](mailto:hanp_elf@163.com); Prof. Yufeng Yan, E-mail address: [Yanyufeng@dingtalk.com](mailto:Yanyufeng@dingtalk.com)


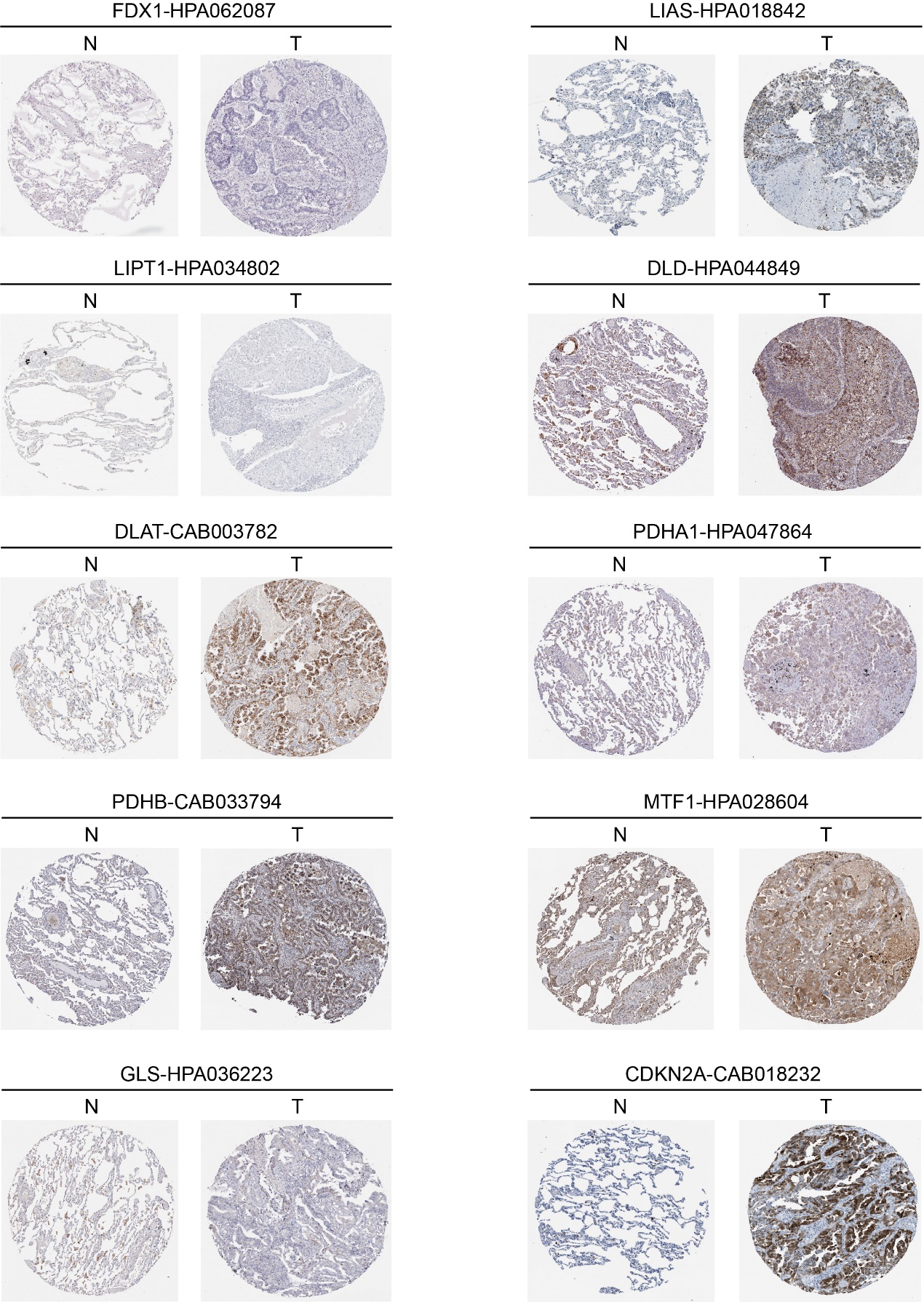


**Figure S1. Translational level validation of 10 cuproptosis-associated genes using the Human Protein Atlas.**


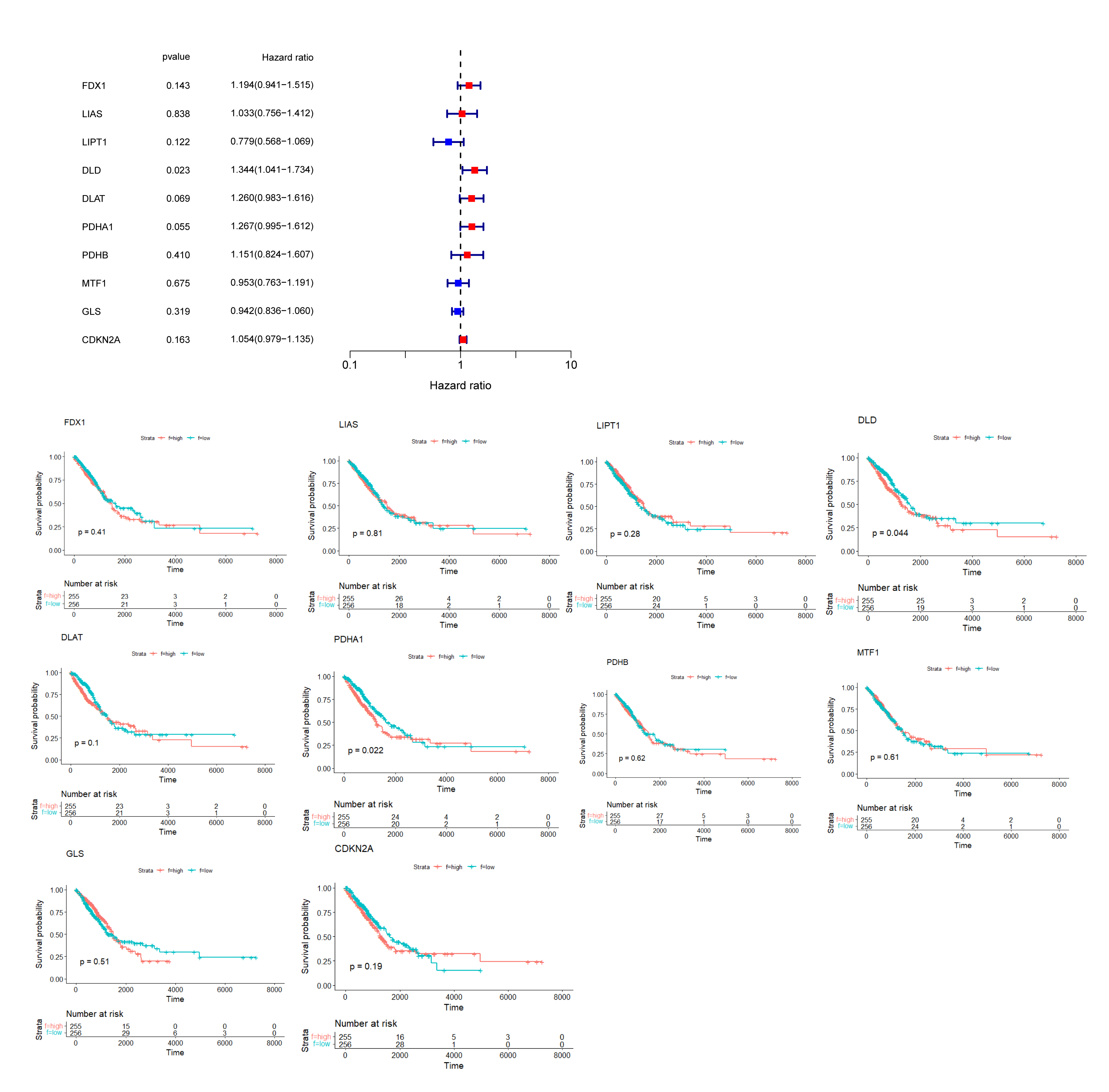


**Figure S2. Univariate Cox regression and Kaplan-Meier curves of 10 cuproptosis-associated genes in TCGA cohort.**


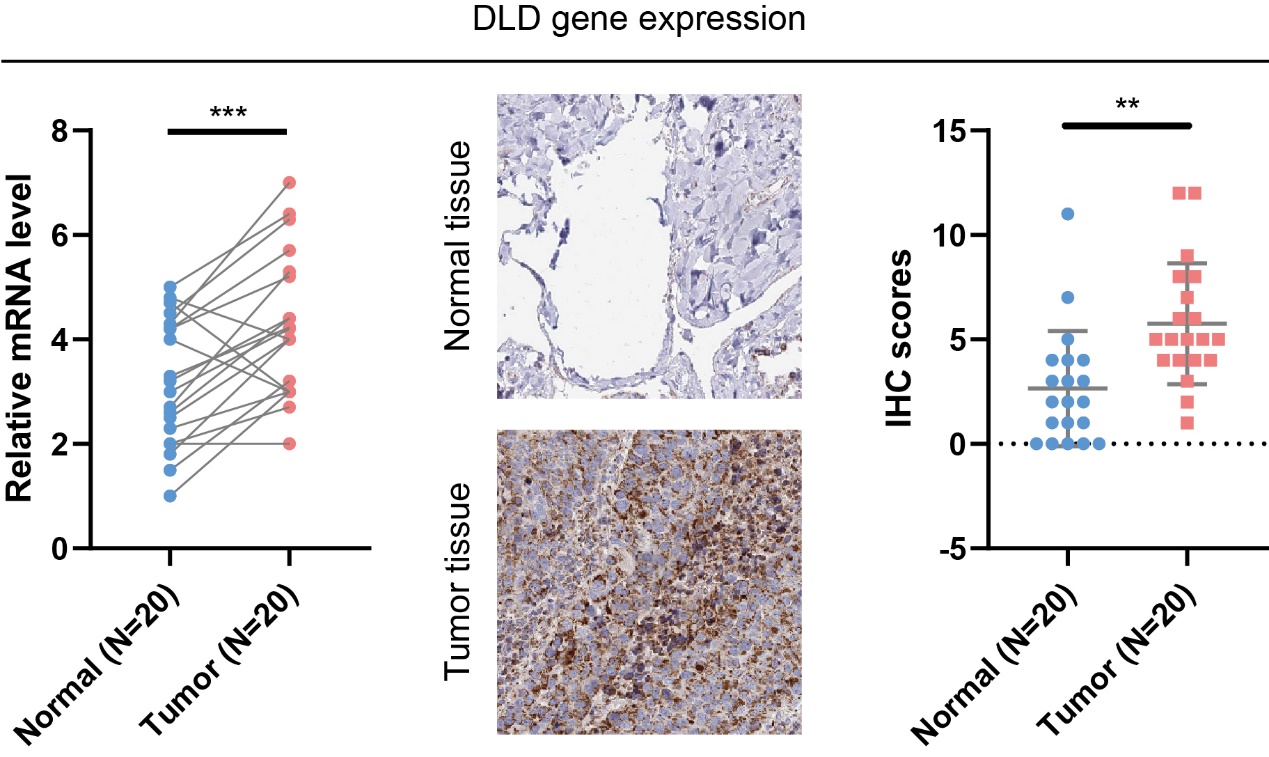


**Figure S3. RT-PCR and immunohistochemistry results showed that the expression level of DLD gene in lung adenocarcinoma patients was higher than that in normal tissues. * indicating P value < 0.05; ** indicating P value <0.01; *** indicating P value <0.001.**

**
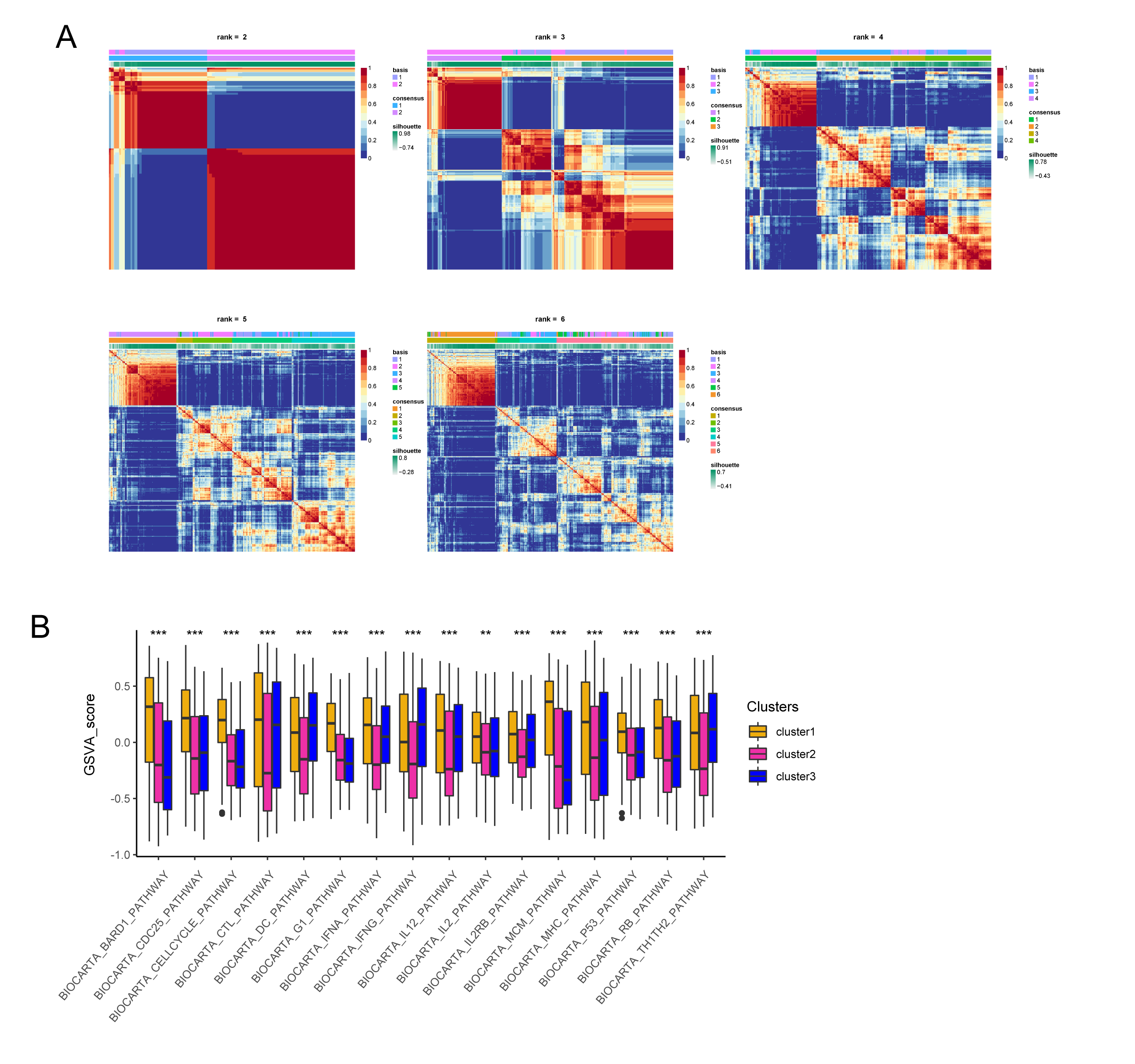
**

**Figure S4. Cuproptosis-associated classifications in lung adenocarcinoma, related to Figure 2.**

**(A)** Heatmap representation of consensus clustering for cuprotosis-related genes in TCGA cohort with cluster numbers from 2 to 6. **(B)** Boxplot showed the GSVA score of pathway activities. Statistical difference is identified by Kruskal-Wallis H test, * indicating P value < 0.05; ** indicating P value <0.01; *** indicating P value <0.001.


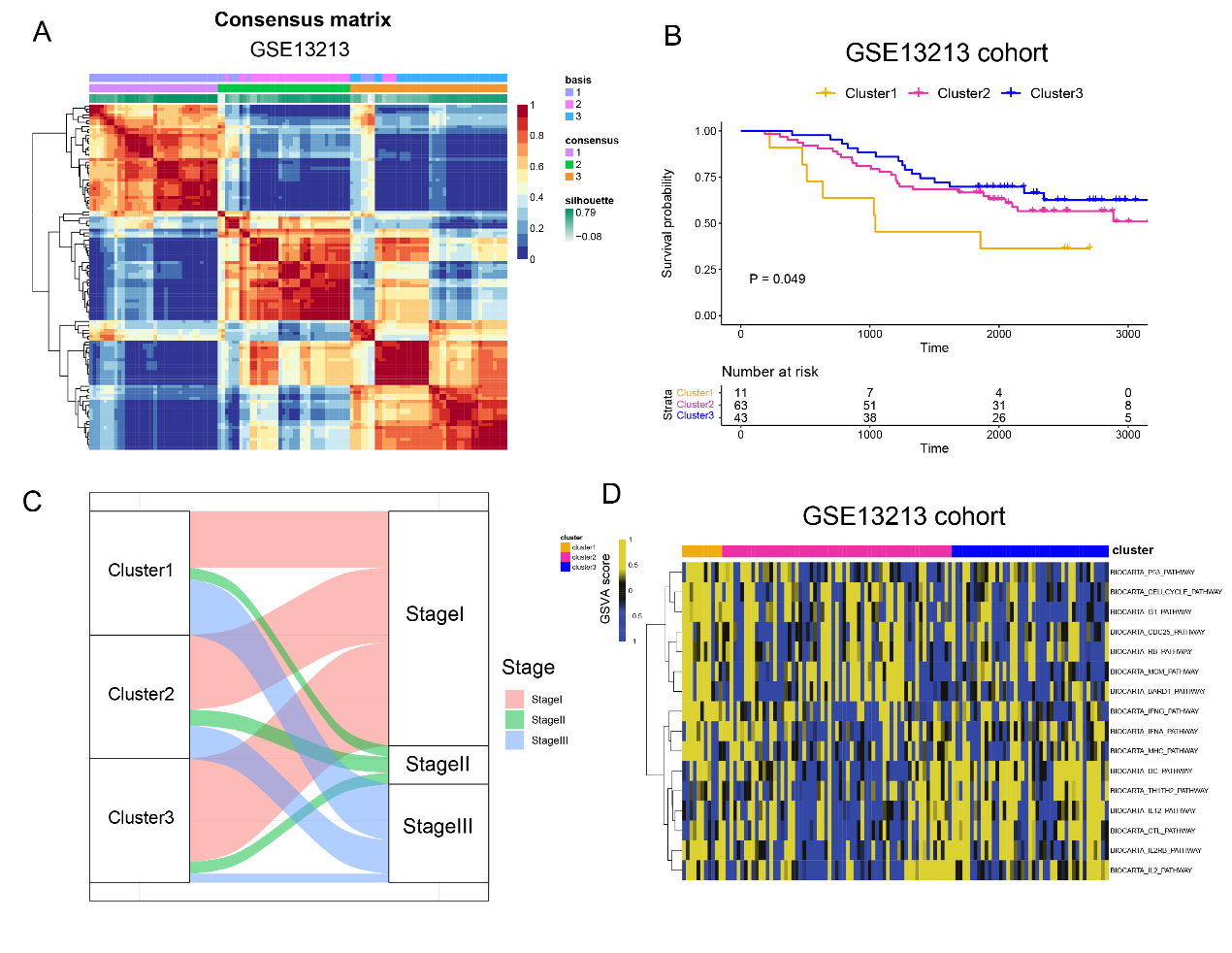


**Figure S5. Cuproptosis-associated classifications in lung adenocarcinoma in GSE13213 cohort.**

(**A**) Non-negative Matrix Factorization (NMF) rank survey was shown. The optimal number of clusters: rank=3. (**B**) K-M survival plots according to NMF clusters. P value was determined by the log-rank test. (**C**) The distribution plot shows the composition of clinicopathological features of three NMF clusters. (**D**) Corresponding pathway activities of three NMF clusters.


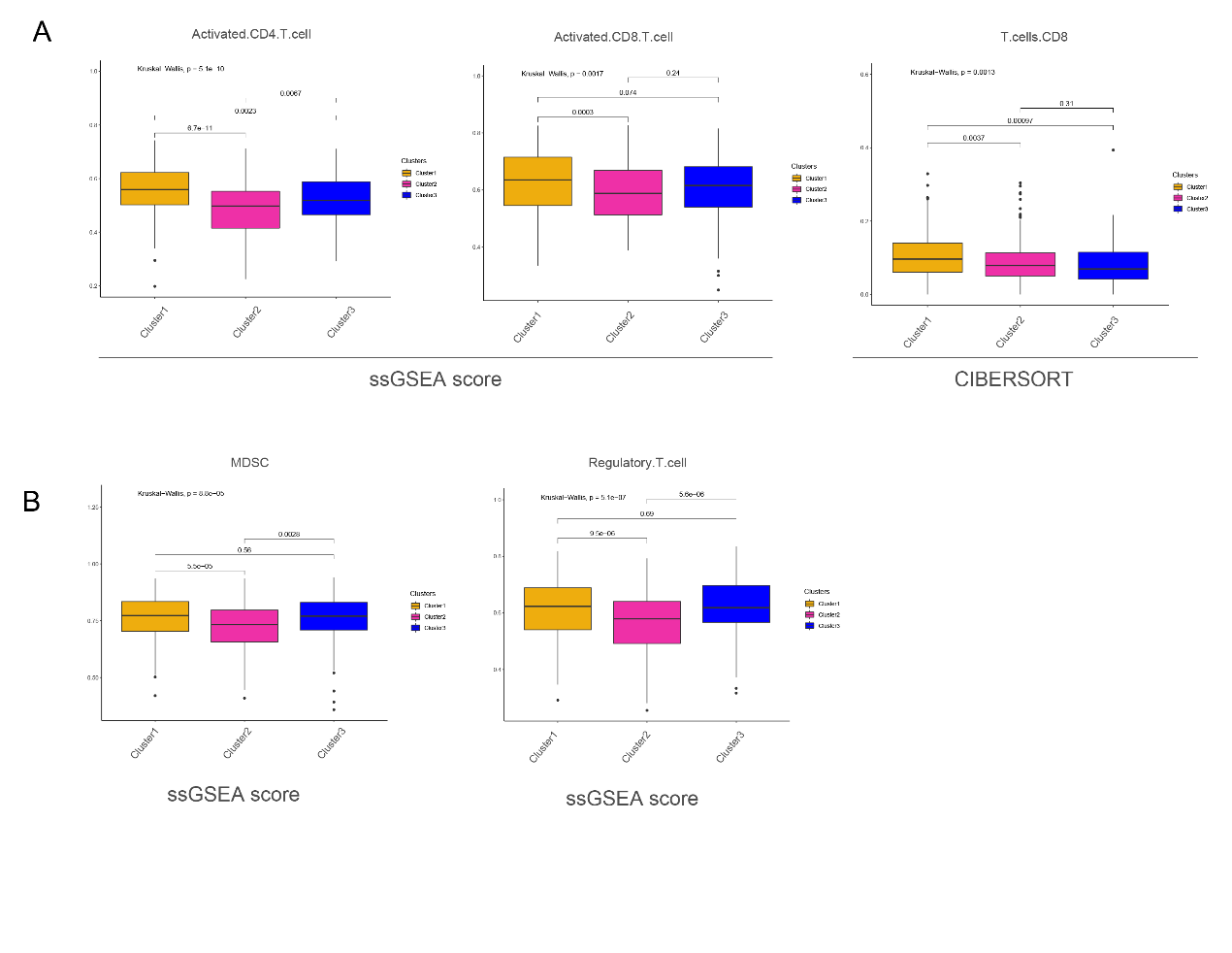


**Figure S6. Comparisons between two groups among three cuproptosis-associated clusters.** (**A**) Anti-tumor-related immune cells (activated CD4/8^+^ T cells and CD8^+^ T cells) were predominantly enriched in cluster1. (**B**) MDSCs and regulatory T cells were mainly enriched in cluster1.


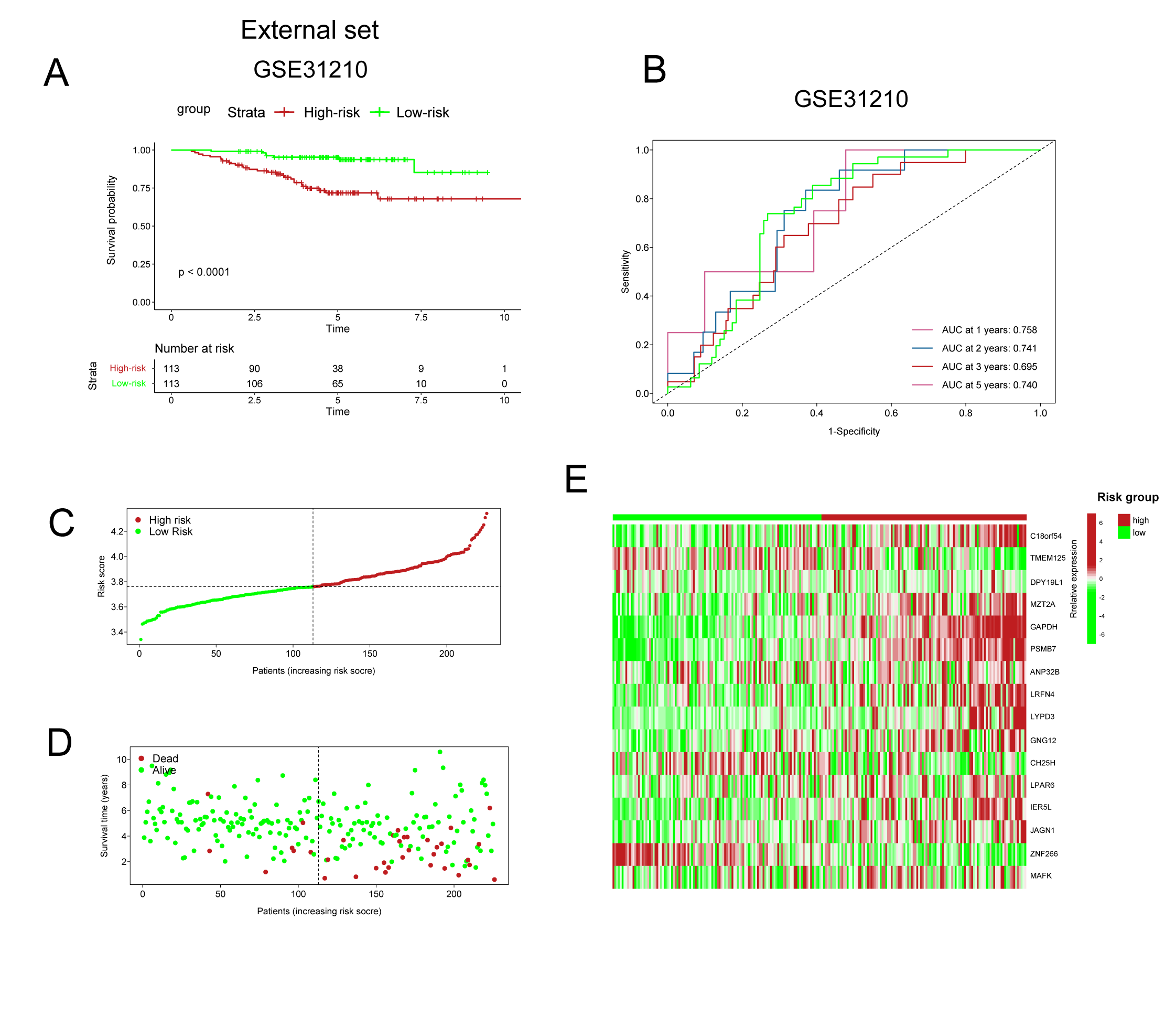


**Figure S7. Development of cuproptosis-associated risk score, related to Figure 4.**

**(A)** Kaplan–Meier analysis of the survival rate between the two groups in TCGA cohort. **(B)** ROC curves to predict the sensitivity and specificity of 1-, 2-, 3-, and 5-year survival according to the cuproptosis-associated gene_score in TCGA cohort. **(C-D)** Ranked dot plots showing the cuproptosis-associated gene_score distribution. **(E)** Differences in the expression of 10 genes between two risk-groups.


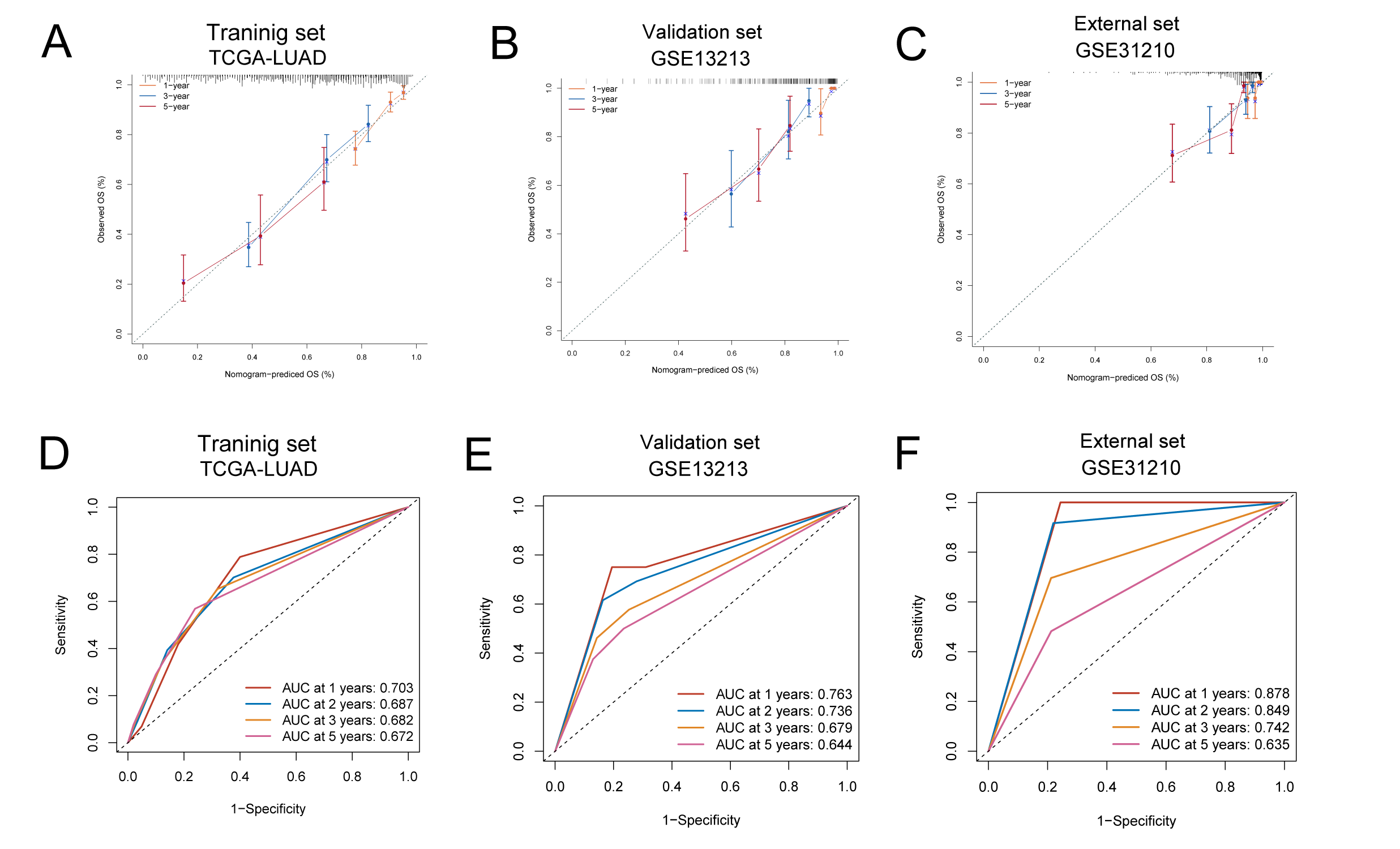


**Figure S8. Constructing a nomogram based on cuproptosis-associated gene score, related to Figure 7.**

**(A-C)** Calibration curves of the nomogram for predicting of 1-, 3-, and 5-year survival rates in the TCGA, validation, and external validation cohort. **(D-F)** ROC curves for predicting the 1-, 2-, 3-, and 5-years, ROC curves in the training (TCGA), validation (GSE13213) cohort, and external cohort (GSE31210).
